# Integrative Multi-Omics Analysis Reveals Germplasm Advantages and Candidate Mechanisms Underlying High Intramuscular Fat Deposition in Wuhuang Pigs

**DOI:** 10.3390/foods15183338

**Published:** 2026-09-20

**Authors:** Xingyu Wang, Yan Zhu, Linyuan Shen, Xia Li, Jiangwen Li, Li Zhu, Mailin Gan

**Affiliations:** 1 State Key Laboratory of Swine and Poultry Breeding Industry, Sichuan Agricultural University, Chengdu 611130, China; 2022202034@stu.sicau.edu.cn (X.W.);; 2 Farm Animal Germplasm Resources and Biotech Breeding Key Laboratory of Sichuan Province, Sichuan Agricultural University, Chengdu 611130, China; 3 Key Laboratory of Livestock and Poultry Multi-Omics, Ministry of Agriculture and Rural Affairs, College of Animal Science and Technology, Sichuan Agricultural University, Chengdu 611130, China; 4 Molecular Breeding Laboratory of National Pig Breeding Industry Park (Santai County), Mianyang 621100, China

**Keywords:** Wuhuang pig, intramuscular fat, multi-omics integrative analysis, transcriptome, metabolome, gut microbiota

## Abstract

Intramuscular fat (IMF) content is a key indicator of pork eating quality. Wuhuang pigs, a fat-type local breed of Sichuan, exhibit high IMF, but the underlying mechanisms remain unclear. Here, Wuhuang and foreign-breed pigs were compared by integrated phenotypic, transcriptomic, metabolomic and 16S rRNA analyses. Crude fat reached 6.40%, 288% higher than in foreign breeds (*p* < 0.001), with lower dressing percentage, loin-eye area, lean percentage (*p* < 0.01) and smaller muscle fibers (*p* < 0.05). Transcriptomics identified 157 differentially expressed genes (DEGs), with core lipogenic genes (SCD, FASN, DGAT2, and SREBF1) upregulated; metabolomics identified 47 differential metabolites enriched in sphingolipids, fatty acids, acylcarnitines and bile acids. Integrated pathway analysis revealed co-directional gene–metabolite changes in common pathways and an SCD/FASN-centered association network. The gut Firmicutes/Bacteroidetes ratio was elevated (about 3.8 vs. 1.9), and 70 differential genera were enriched in short-chain-fatty-acid producers (e.g., *Bifidobacterium*, *Streptococcus*, and *Lachnospiraceae*) and bile-acid metabolizers (e.g., *Parasutterella*). High IMF may thus be associated with coordinated changes along the “gut microbiota–host metabolism–gene expression–fat deposition” axis, providing candidate genes, metabolites and microbial taxa that may help conserve this local breed and improve pork quality.

## 1. Introduction

Adipose tissue is widely distributed in subcutaneous, visceral, intermuscular and intramuscular sites. Intramuscular fat (IMF) is deposited in the perimysium and endomysium of skeletal muscle, and its content and composition directly affect eating quality indicators such as flavor, tenderness, juiciness and shear force [1,2]. In pork production, an appropriate IMF content (above 2–3%) is an important prerequisite for the production of premium high-quality pork, and deciphering the molecular regulatory mechanisms of IMF deposition is of great significance for the genetic improvement of meat quality traits [3].

The IMF content of modern commercial pig breeds generally decreases after directional selection for lean meat percentage, accompanied by a certain degradation of meat flavor and quality [4,5]. In contrast, Chinese local pig breeds generally possess higher IMF content and are valuable genetic resources for studying the molecular mechanisms of fat deposition [1]. Wuhuang pigs, commonly known as “Wuhuang old trough pigs”, are a fat-type, large, late-maturing local pig breed unique to Yanjiang District, Ziyang City, Sichuan Province, characterized by a large body size, tolerance to coarse feed, strong reproductive capacity, high disease resistance and a docile temperament. However, in recent years, the population of Wuhuang pigs has continued to decline, and the conservation of its germplasm resources faces a severe situation. According to the Third National Survey of Livestock and Poultry Genetic Resources, only 2811 Wuhuang pigs (including 1802 breeding sows and 34 breeding boars) remained in Ziyang at the time of the survey, and the breed is currently maintained mainly through in situ conservation programs [6]. Although previous studies have preliminarily characterized its genetic structure and phenotypic traits, the molecular mechanisms underlying its high IMF formation still lack systematic investigation [7,8].

IMF deposition is a complex biological process involving multiple steps, including adipocyte differentiation, fatty acid synthesis and transport, and triglyceride synthesis and storage [9,10]. The development of high-throughput sequencing and mass spectrometry technologies has made integrative multi-omics analysis a powerful tool for deciphering the molecular mechanisms of complex economic traits [11,12]. Meanwhile, gut microbiota participate in the regulation of host lipid metabolism and fat deposition through the “microbiota–gut–adipose” axis [13,14,15,16], in which short-chain-fatty-acid-producing families such as the *Lachnospiraceae* and *Ruminococcaceae*, together with bile-acid-transforming genera such as *Bacteroides*, modulate fatty-acid oxidation, de novo lipogenesis and triglyceride storage, but how gut microbiota influence skeletal muscle lipid metabolism through metabolic signals remains to be elucidated.

In this study, Wuhuang pigs were used as the research subjects and foreign breeds as controls. Through phenotypic measurement, transcriptome sequencing, untargeted metabolomics and 16S rRNA sequencing, the differences between the two groups in carcass performance, meat quality traits, gene expression, metabolite composition and gut microbial community were systematically compared, and integrative multi-omics analysis was employed to explore the molecular mechanisms of high IMF formation in Wuhuang pigs, thereby providing a theoretical basis for the conservation and utilization of Wuhuang pig germplasm resources and the improvement of meat quality traits in local pig breeds.

## 2. Materials and Methods

### 2.1. Experimental Animals and Sample Collection

All pigs used in this study were female and were 150 days old at slaughter. Wuhuang pigs (denoted “WHZ”) were obtained from a pig breeding company in southwest China, and the controls (denoted “WZZ”) were commercial Duroc × Landrace × Yorkshire (DLY) crossbred pigs from our research group. All animals came from a single cohort raised under identical conditions and slaughtered on the same day. Fecal samples were collected per rectum from 13 WHZ and 10 WZZ pigs one day before slaughter. Because the WHZ pigs represent a valuable and scarce local breed resource and the amount of tissue available for destructive assays was limited, six WHZ and 14 WZZ pigs were selected for slaughter according to the requirements and costs of the individual assays, and each assay was then performed on the animals specified for it: carcass traits (n = 6 WHZ versus n = 14 WZZ), meat-quality traits (n = 6 WHZ versus n = 7 WZZ), transcriptome sequencing (n = 3 per group), untargeted metabolomics (n = 6 WHZ versus n = 9 WZZ), and fecal 16S rRNA sequencing (n = 12 WHZ versus n = 10 WZZ). The number of animals therefore differs among assays because each assay followed its own design and was performed on the subset of animals for which the corresponding sample was available and passed the assay-specific quality-control criteria; for fecal 16S rRNA sequencing, one WHZ animal (WHZ13) was excluded as a quality-control outlier, leaving n = 12 WHZ versus n = 10 WZZ (Supplementary Table S1); in every assay, the individual animal served as the biological replicate and the statistical unit. All animals were clinically healthy, with no clinical signs of genetic or infectious disease, and were immunized according to the farm’s standard vaccination program; no antibiotics were administered within one month before sampling. Both groups were raised under the same standardized housing and management (slatted-floor pens, natural ventilation, ad libitum drinking water and the same corn–soybean-meal diet formulated to NRC (2012) recommendations [17]) and were slaughtered at the same age (150 days) under identical lairage, stunning and slaughter procedures in the same abattoir. Fecal samples were stored at −80 °C. Immediately after slaughter, longissimus dorsi muscle samples were collected, snap-frozen in liquid nitrogen and stored at −80 °C for meat quality determination, transcriptome and metabolome analyses.

### 2.2. Carcass Performance and Meat Quality Determination

According to the agricultural industry standard NY/T 825-2004 [18], the body weight before slaughter, carcass weight, dressing percentage, average backfat thickness, loin eye area, lean meat percentage, skin and fat percentage and bone percentage were determined. Meat quality indicators including pH45min, pH24h, meat color (L45min, L24h), pressing loss, cooking yield, shear force and crude fat content (Soxhlet extraction method) were also determined. Specifically, pH45min and pH24h denote muscle pH measured at 45 min and 24 h postmortem, and L45min and L24h denote CIE L* (lightness) recorded at 45 min and 24 h postmortem; pressing loss reflects water-holding capacity and shear force reflects tenderness. Carcass traits were compared between WZZ (n = 14) and WHZ (n = 6), and meat-quality traits between WZZ (n = 7) and WHZ (n = 6). Throughout this paper, intramuscular fat (IMF) denotes the total lipid deposited within the longissimus dorsi muscle, and the crude fat content determined by the Soxhlet extraction method (g per 100 g fresh muscle) is used as its quantitative chemical measure; “crude fat content” and “IMF content” therefore refer to the same intramuscular measurement and are distinct from intermuscular and subcutaneous fat.

### 2.3. Transcriptome Sequencing Analysis

Total RNA was extracted from the longissimus dorsi muscle using the Trizol method (TRIzol reagent; Invitrogen, Carlsbad, CA, USA), with three biological replicates per group (n = 3). After quality inspection with a NanoDrop 2000 spectrophotometer (Thermo Fisher Scientific, Waltham, MA, USA) and an Agilent 2100 Bioanalyzer (Agilent Technologies, Santa Clara, CA, USA), cDNA libraries were constructed using the Illumina TruSeq Stranded mRNA Library Preparation Kit (Illumina, San Diego, CA, USA) and sequenced on the Illumina NovaSeq 6000 platform (Illumina, San Diego, CA, USA). Data were quality-controlled with fastp, aligned to the pig reference genome (Sus scrofa 11.1) with HISAT2 and quantified with featureCounts. Per-library sequencing quality (clean-read Q30, GC content and unique-mapping rate) was inspected and all libraries passed the standard thresholds; raw read counts were normalized by the DESeq2 median-of-ratios method (and variance-stabilized for multivariate plotting) before PLS-DA. Differentially expressed genes were screened with DESeq2 (|log2FC| > 1 and padj < 0.05), and GO and KEGG (Kyoto Encyclopedia of Genes and Genomes) [19] enrichment analyses were performed using the clusterProfiler package.

### 2.4. Untargeted Metabolomics Analysis

Longissimus dorsi muscle samples (n = 6 WHZ and n = 9 WZZ) were extracted with 80% methanol and subjected to untargeted metabolomics analysis by UHPLC-MS/MS (Shimadzu Nexera X2 LC-30AD (Shimadzu Corporation, Kyoto, Japan) and AB SCIEX Triple TOF 6600 (SCIEX, Framingham, MA, USA)). Features detected in fewer than 80% of samples were removed, missing values were imputed by the group median, and the retained matrix was normalized to total ion intensity, log2(x + 1)-transformed and Pareto-scaled (mean-centered) before multivariate analysis. Data were processed with Progenesis QI, and metabolites were identified by matching against the Human Metabolome Database (HMDB) [20], the METLIN Metabolite and Chemical Entity Database (METLIN) [21] and the Kyoto Encyclopedia of Genes and Genomes (KEGG) [19] databases. To assess overall differences in metabolite profiles between groups, orthogonal partial least squares discriminant analysis (OPLS-DA, NIPALS) was performed and validated by leave-one-out cross-validation (LOOCV); the final model comprised one predictive and one Y-orthogonal component (the Y-orthogonal component raised cross-validated Q2 from 0.347 to 0.732, with no further gain at two components). The model yielded R2X = 0.555, R2Y = 0.974 and Q2 = 0.732, with an LOOCV classification accuracy of 93.3% (14/15) and AUC = 0.981, and robustness against over-fitting was confirmed by a 1000-permutation test (permutation *p* = 0.002 for Q2 and 0.006 for R2Y; Q2 regression intercept = −0.489). Differential metabolites were screened using the criteria of VIP > 1 and *p* < 0.05. Supplementary Figure S1 shows the validation of this model: (Figure S1A) the unsupervised PCA score plot, in which the WZZ and WHZ samples separate clearly along the principal axes with tight within-group clustering of biological replicates; (Figure S1B) the 1000-permutation test, in which the observed Q2 exceeded the permuted null distribution (*p* = 0.002; Q2 intercept = −0.489), confirming that the separation is genuine rather than over-fitted; and (Figure S1C) the OPLS-DA S-plot, highlighting the key differential metabolites (VIP ≥ 1) driving the separation and their abundance trends in the two groups.

### 2.5. 16S rRNA Sequencing Analysis

Genomic DNA of fecal microorganisms was extracted using the CTAB method from the fecal samples of 13 WHZ and 10 WZZ pigs; DNA concentration and purity were verified with a NanoDrop spectrophotometer and 1% agarose-gel electrophoresis, and samples with a DNA concentration below 20 ng/μL, an A260/A280 ratio outside 1.8–2.0, or evident degradation on the gel were re-extracted or excluded before library construction. The V3–V4 region of the 16S rRNA gene was amplified with the barcoded primers 515F (5′-GTGCCAGCMGCCGCGGTAA-3′) and 806R (5′-GGACTACHVGGGTWTCTAAT-3′); the amplicons were purified, quantified and pooled, then paired-end sequenced (2 × 250 bp) on the Illumina NovaSeq 6000 platform. ASVs were obtained by denoising with QIIME2 (DADA2), and taxonomic assignment was performed against the SILVA reference database; individual samples were first rarefied to equal sequencing depth and then screened by pre-specified quality-control criteria based on alpha-diversity indices: samples with fewer than 600 observed ASVs, a Shannon index below 5.5, or a dominance index above 0.20 were considered low-diversity outliers and excluded from downstream analysis. On this basis, one WHZ sample (WHZ13; 594 observed ASVs, Shannon 4.82, dominance 0.233) was excluded, as four of its alpha-diversity indices lay outside ±3 SD of the group mean (two exceeded ±6 SD); the sample-level alpha-diversity values for all animals are given in Supplementary Table S1. Retaining this sample in a sensitivity analysis did not change the group-level directions of the differential genera or the beta-diversity results. Raw read depth did not differ between groups (Wilcoxon *p* > 0.05). Alpha/beta diversity and species composition were then analysed. Differential genera were tested with the Wilcoxon rank-sum test and Benjamini–Hochberg false-discovery-rate (FDR) correction, with effect size reported as log2 fold change; genera with nominal *p* < 0.05 are displayed in the figures. Between-group beta-diversity differences were tested by PERMANOVA (999 permutations) based on Bray–Curtis distances using the vegan package in R.

### 2.6. Real-Time Quantitative Polymerase Chain Reaction (RT-qPCR)

Total RNA was extracted from LT with RNAiso Plus (TaKaRa, Dalian, China), and concentration and purity were checked spectrophotometrically. Equal RNA inputs were converted to cDNA with the PrimeScript RT reagent kit (TaKaRa). Quantitative PCR was performed using SYBR Premix Ex Taq kit (TaKaRa). The amplification was performed according to the manufacturers’ instructions. For the six genes in this study, primers were designed using the NCBI online tool (https://www.ncbi.nlm.nih.gov/ (accessed on 10 August 2026)), and all primer sequences were verified for specificity using BLAST+ (version 2.16.0), as shown in Table 1. Relative transcript abundance was calculated using the 2^−ΔΔCt^ method with GAPDH as the reference. Three biological replicates per group (n = 3) were analyzed, each in three technical replicates. Primer specificity was confirmed by a single melting-curve peak and BLAST verification; amplification efficiencies over a five-point standard-curve dilution series were within 90–110% (R^2^ > 0.99). GAPDH was chosen as the reference gene after confirming stable expression across groups, and relative transcript abundance was calculated by the 2^−ΔΔCt^ method from technical-replicate means per animal.

### 2.7. Statistical Analysis

Data analysis was performed using R (4.2.0) and Python (3.10). Normality of each variable was first assessed by the Shapiro–Wilk test and homogeneity of variance by Levene’s test: normally distributed, homoscedastic variables were compared by the two-sided, unpaired Student’s *t*-test (Welch’s *t*-test when variances were unequal), whereas non-normally distributed data were compared by the non-parametric Wilcoxon rank-sum (Mann–Whitney U) test. For all high-dimensional analyses (DEGs, differential metabolites and differential genera), *p*-values were adjusted with the Benjamini–Hochberg false-discovery-rate (FDR) procedure, and effect sizes are reported as log2 fold change. R was used for the statistical tests, OPLS-DA (ropls package), alpha/beta diversity and PERMANOVA analyses (vegan package); Python was used for data integration (KEGG pathway intersection and the gene–metabolite nine-quadrant analysis), network construction and heatmaps; and GraphPad Prism 6.0 (GraphPad Software, Boston, MA, USA) was used for bar charts. Integrative multi-omics analyses included the KEGG pathway intersection, gene–metabolite nine-quadrant plots and the construction of an integrated association (candidate pathway–gene–metabolite) network with Cytoscape v3.9.1. Because the transcriptomic and metabolomic assays were performed on different individuals of the same slaughter cohort, cross-omics integration was conducted at the group level rather than by per-animal paired correlation: KEGG pathways enriched (adjusted *p* < 0.05) in both the DEG and differential-metabolite sets were intersected, and within each shared pathway each DEG was paired with every co-annotated differential metabolite, yielding 117 gene–metabolite pairs; the nine-quadrant plot then classified these pairs by the sign and magnitude of their group-level log2 fold changes to assess directional concordance. OPLS-DA was performed using the ropls R package with leave-one-out cross-validation (one predictive and one Y-orthogonal component).

## 3. Results

### 3.1. Carcass Characteristics and Meat Quality of Wuhuang Pigs

As shown in Table 2, compared with foreign-breed pigs, the body weight before slaughter, carcass weight, dressing percentage, loin eye area and lean meat percentage of Wuhuang pigs were significantly decreased (*p* < 0.01), while the average backfat thickness, skin and fat percentage and bone percentage were significantly increased (*p* < 0.05), exhibiting typical characteristics of a fat-type pig breed.

The meat quality results are shown in Table 3: the L45min, L24h and pressing loss of Wuhuang pigs were significantly decreased (*p* < 0.05), and the crude fat content was significantly increased (*p* < 0.001, an increase of 288%); pH45min, pH24h, cooking yield and shear force showed no significant differences (*p* > 0.05). The significantly lower pressing loss and numerically lower shear force indicate improved water-holding capacity and tenderness potential of Wuhuang pork.

### 3.2. Morphological Characteristics of Skeletal Muscle of Wuhuang Pigs

**Figure 1:**
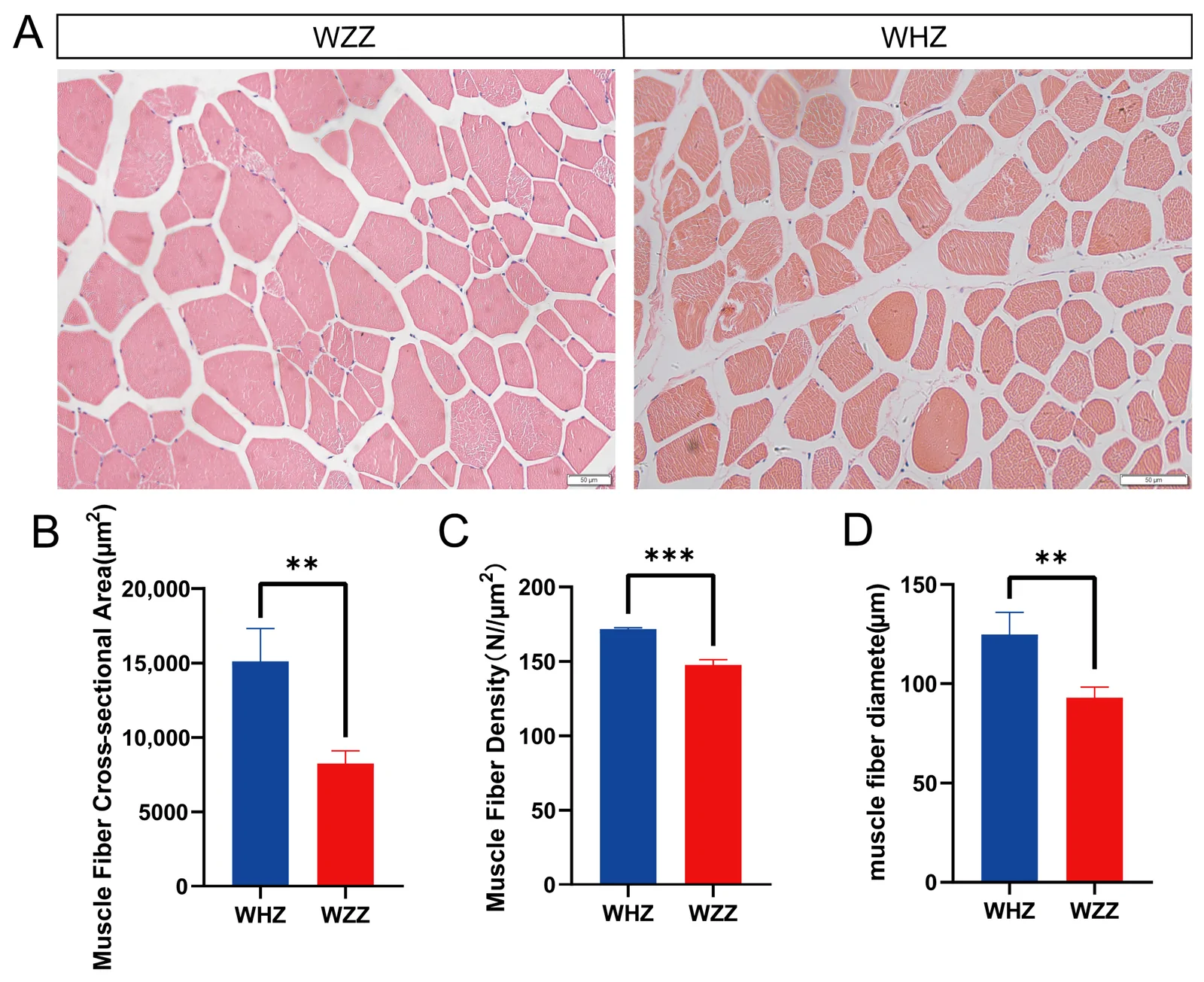
Histological staining of the longissimus dorsi muscle with hematoxylin and eosin (HE) and corresponding histomorphometric analysis. (**A**) HE staining of the longissimus dorsi muscle; (**B**) muscle fiber cross-sectional area; (**C**) muscle fiber density; (**D**) muscle fiber diameter. Statistical significance: ** *p* < 0.01, *** *p* < 0.001. WHZ: Wuhuang pig; WZZ: foreign-breed pig (commercial crossbred pig, Duroc × Landrace × Yorkshire, DLY). Magnification and scale bars are indicated in the HE panels; for each animal, five randomly selected microscopic fields were measured and averaged, and group comparisons used the per-animal mean (n = 6 WHZ vs. n = 7 WZZ) as the statistical unit—individual muscle fibers were not treated as independent biological replicates.

HE staining showed that Wuhuang pigs had smaller and more dispersed muscle fiber bundles with more obvious intramuscular fat deposition (Figure 1A); quantitative analysis revealed that the muscle fiber cross-sectional area (Figure 1B), density (Figure 1C) and diameter (Figure 1D) were all significantly lower than those of foreign-breed pigs (*p* < 0.05), suggesting obvious differences in the skeletal muscle development patterns between the two groups.

### 3.3. mRNA Identification and Differential Expression Analysis

**Figure 2:**
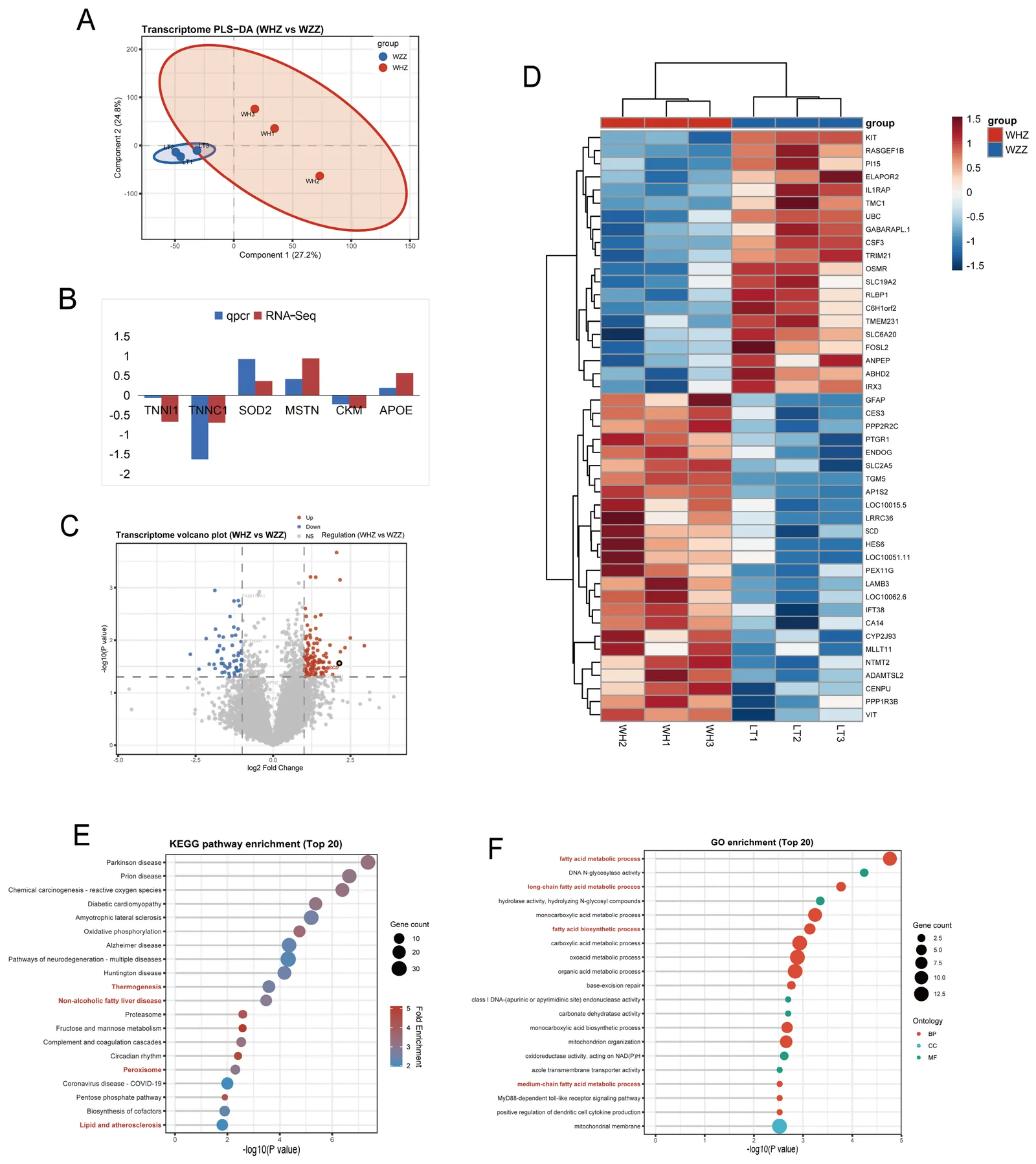
Comparative transcriptomic analysis of longissimus dorsi muscle between Wuhuang pigs and foreign-breed pigs. (**A**) PLS-DA score plot of transcriptomic samples; (**B**) RT-qPCR validation of selected genes, consistent with the RNA-seq results; (**C**) volcano plot of DEGs (red, upregulated; blue, downregulated; |log2FC| ≥ 1 and *p*adj < 0.05); (**D**) clustering heatmap of DEGs; (**E**) KEGG pathway enrichment analysis (Top 20); and (**F**) GO functional enrichment analysis (Top 20). WHZ: Wuhuang pig; WZZ: foreign-breed pig (commercial crossbred pig, Duroc × Landrace × Yorkshire, DLY). Transcriptome sequencing was performed on n = 3 WHZ and n = 3 WZZ animals.

RNA-seq identified a total of 13,660 expressed genes. PLS-DA analysis showed that the samples within each group clustered closely while the two groups were clearly separated, indicating significant differences between the transcriptomes of the two groups (Figure 2A); the PLS-DA was performed on DESeq2-normalized, variance-stabilized expression and was cross-validated. A total of 157 DEGs (89 upregulated and 68 downregulated) were identified using the criteria of |log2FC| > 1 and *p*adj < 0.05 (Figure 2C). The qPCR validation results were consistent with the RNA-seq trends (Figure 2B). The clustering heatmap clearly distinguished the samples of the two groups (Figure 2D). GO enrichment analysis showed that the DEGs were significantly enriched in biological processes such as lipid metabolic process, fatty acid biosynthesis and adipocyte differentiation (Figure 2F); among them, fatty-acid metabolic process was the leading biological process (11 genes, adjusted *p* = 0.013), followed by long-chain fatty-acid metabolic process (4 genes) and fatty-acid biosynthetic process (6 genes), and KEGG enrichment analysis showed significant enrichment in peroxisome, thermogenesis, non-alcoholic fatty liver disease and PPAR signaling pathways (Figure 2E) [22]. The upregulated genes included known lipogenesis- and lipid metabolism-related genes such as stearoyl-CoA desaturase (SCD), fatty-acid synthase (FASN), diacylglycerol O-acyltransferase 2 (DGAT2), perilipin 1 (PLIN1), adiponectin C1Q and collagen-domain-containing (ADIPOQ), thyroid-hormone-responsive protein (THRSP), ELOVL fatty-acid elongase 6 (ELOVL6) and sterol-regulatory-element-binding transcription factor 1 (SREBF1).

### 3.4. Metabolite Identification and Differential Expression Analysis

**Figure 3:**
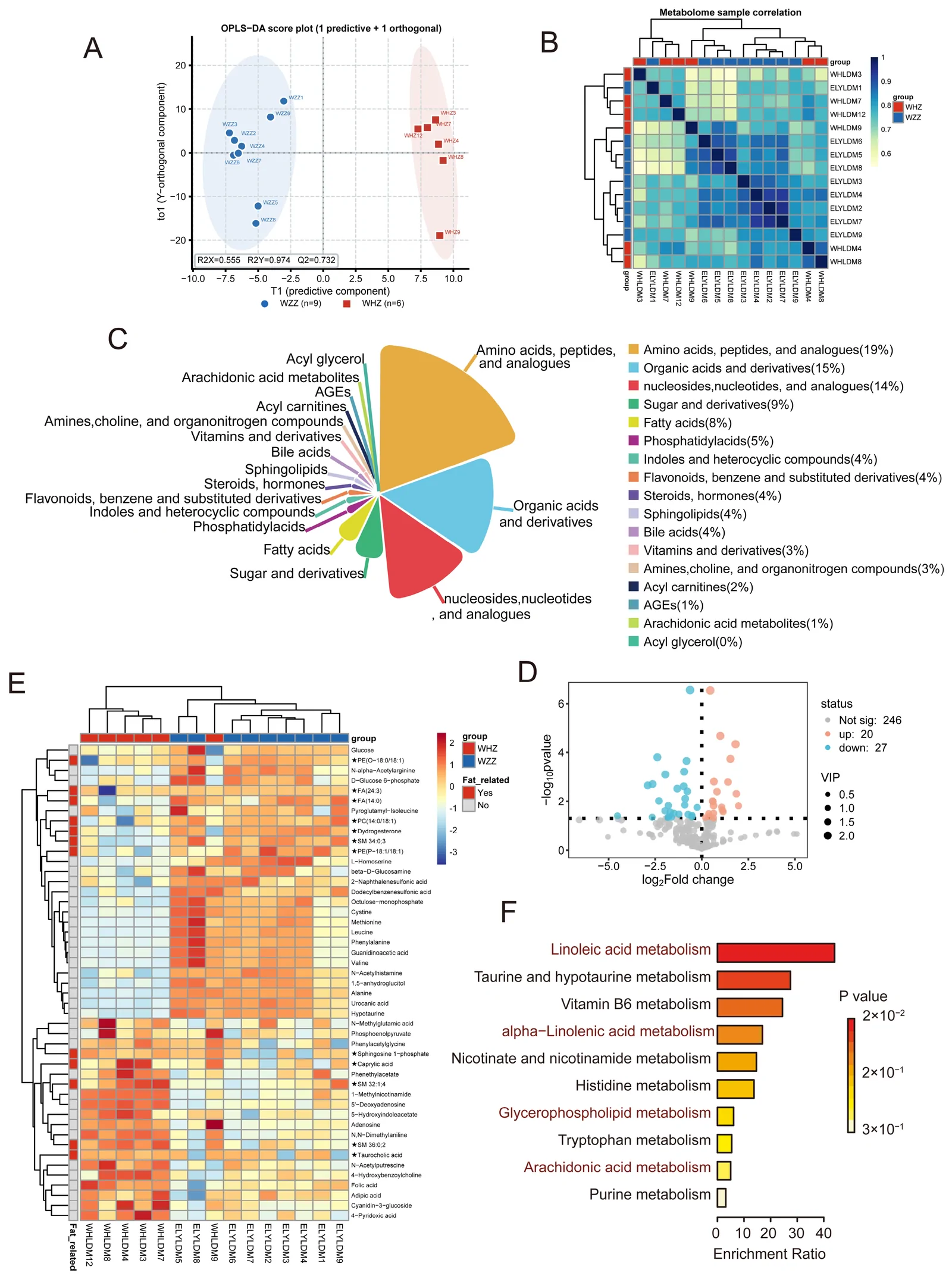
Metabolomic analysis of longissimus dorsi muscle between Wuhuang pigs and foreign-breed pigs. (**A**) OPLS-DA score plot of the metabolome (R2Y = 0.974, Q2 = 0.732); (**B**) heatmap of Pearson correlations between samples; (**C**) proportions of the chemical classes of the 293 identified metabolites (17 classes); (**D**) volcano plot of differential metabolites (point size indicates the VIP value; red, upregulated; blue, downregulated); (**E**) heatmap of lipid-related differential metabolites (asterisks indicate fat-related metabolites); and (**F**) KEGG pathway enrichment analysis of differential metabolites. WHZ: Wuhuang pig; WZZ: foreign-breed pig (commercial crossbred pig, Duroc × Landrace × Yorkshire, DLY). Untargeted metabolomics was performed on n = 6 WHZ and n = 9 WZZ animals.

OPLS-DA analysis showed clear separation of the metabolic profiles between the two groups (R2Y = 0.974, Q2 = 0.732; 1000-permutation test *p* = 0.002 for Q2; cross-validated accuracy 93.3% (14/15), AUC = 0.981) (Figure 3A), and the inter-sample correlation analysis indicated good experimental reproducibility (Figure 3B). A total of 293 metabolites (17 classes, Figure 3C) and 47 differential metabolites (32 upregulated and 15 downregulated, Figure 3D) were identified, among which lipid metabolites such as sphingolipids, fatty acids, acylcarnitines, bile acids and glycerophospholipids were generally upregulated in Wuhuang pigs (Figure 3E); for example, sphingosine-1-phosphate (log2FC = 1.97, VIP = 1.50), SM 36:0;2 (log2FC = 1.00, VIP = 2.26), taurocholic acid (log2FC = 1.10, VIP = 1.58) and caprylic acid were higher in WHZ, whereas SM 34:0;3 (log2FC = −2.39, VIP = 2.20) and several phosphatidylcholines/phosphatidylethanolamines were lower, consistent with previous reports on the metabolomic differences in meat quality among different pig breeds [23]. KEGG enrichment analysis showed that the differential metabolites were mainly enriched in lipid metabolic pathways such as sphingolipid metabolism, glycerophospholipid metabolism, steroid hormone biosynthesis, primary bile acid biosynthesis and fatty acid biosynthesis (Figure 3F), suggesting that the metabolic activities related to lipid synthesis and deposition are more active in the muscle of Wuhuang pigs.

### 3.5. Integrative Analysis of RNA-Seq and Untargeted Metabolomics

**Figure 4:**
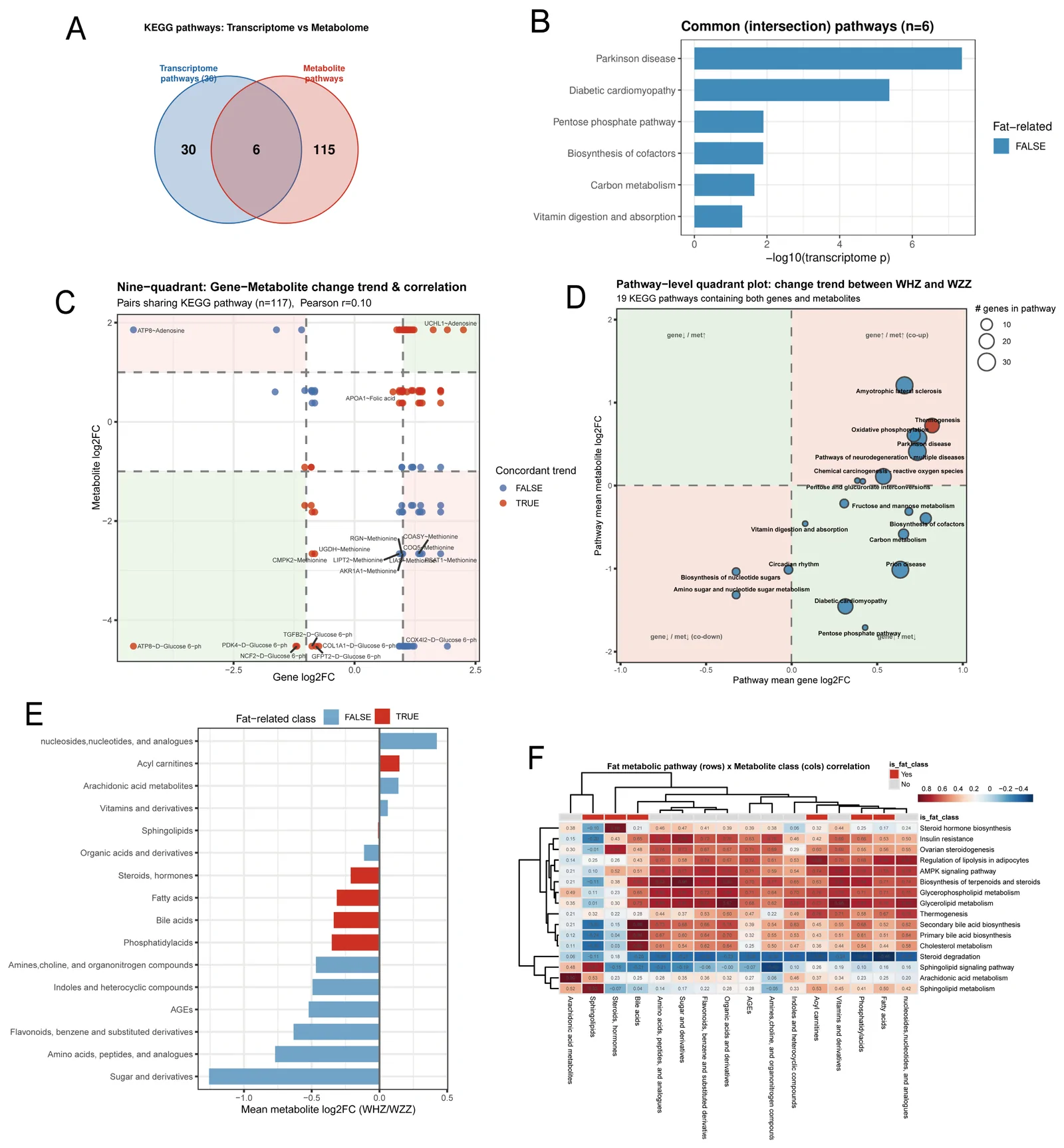
Integrative analysis of transcriptome and metabolome. (**A**) Venn diagram of differential KEGG pathways between the transcriptome (36) and the metabolome (115), showing six common pathways; (**B**) enrichment bar plot of the common pathways; (**C**) gene–metabolite nine-quadrant plot (117 pairs); (**D**) pathway-level quadrant plot; (**E**) bar plot of the mean log2FC of fat-related metabolite classes; (**F**) correlation heatmap between fat metabolic pathways and metabolite classes. WHZ: Wuhuang pig; WZZ: foreign-breed pig (commercial crossbred pig, Duroc × Landrace × Yorkshire, DLY).

The intersection of the DEG pathways (36) and the differential metabolite pathways yielded six common pathways, mainly clustered at nodes such as carbon metabolism, biosynthesis of cofactors and lipid metabolism (Figure 4A,B). Based on the KEGG annotations, the DEGs and differential metabolites were associated by co-annotation to the same KEGG pathway (within each shared pathway, every DEG was paired with each co-annotated differential metabolite), which yielded 117 gene–metabolite pairs in total, and the gene–metabolite nine-quadrant plot showed that most pairs changed in the same direction, suggesting that the changes at the transcriptional level were consistent with the trends in metabolite levels (Figure 4C). The pathway-level quadrant analysis further showed that the gene and metabolite changes of most common pathways were consistent in direction, with the genes and metabolites of fat deposition-related pathways such as thermogenesis showing an overall upward trend in Wuhuang pigs (Figure 4D). Summarized by metabolite chemical class (Figure 4E), the mean log2FC of fat-related metabolite classes such as fatty acids, sphingolipids, bile acids and steroids was generally positive in Wuhuang pigs; the correlation analysis between fat metabolic pathways and the above lipid metabolite classes showed that they were mostly positively correlated (Figure 4F), indicating that the enhanced activity of lipogenic pathways accompanies the accumulation of lipid metabolites, which is consistent with the stronger fat deposition capacity of Wuhuang pigs.

**Figure 5:**
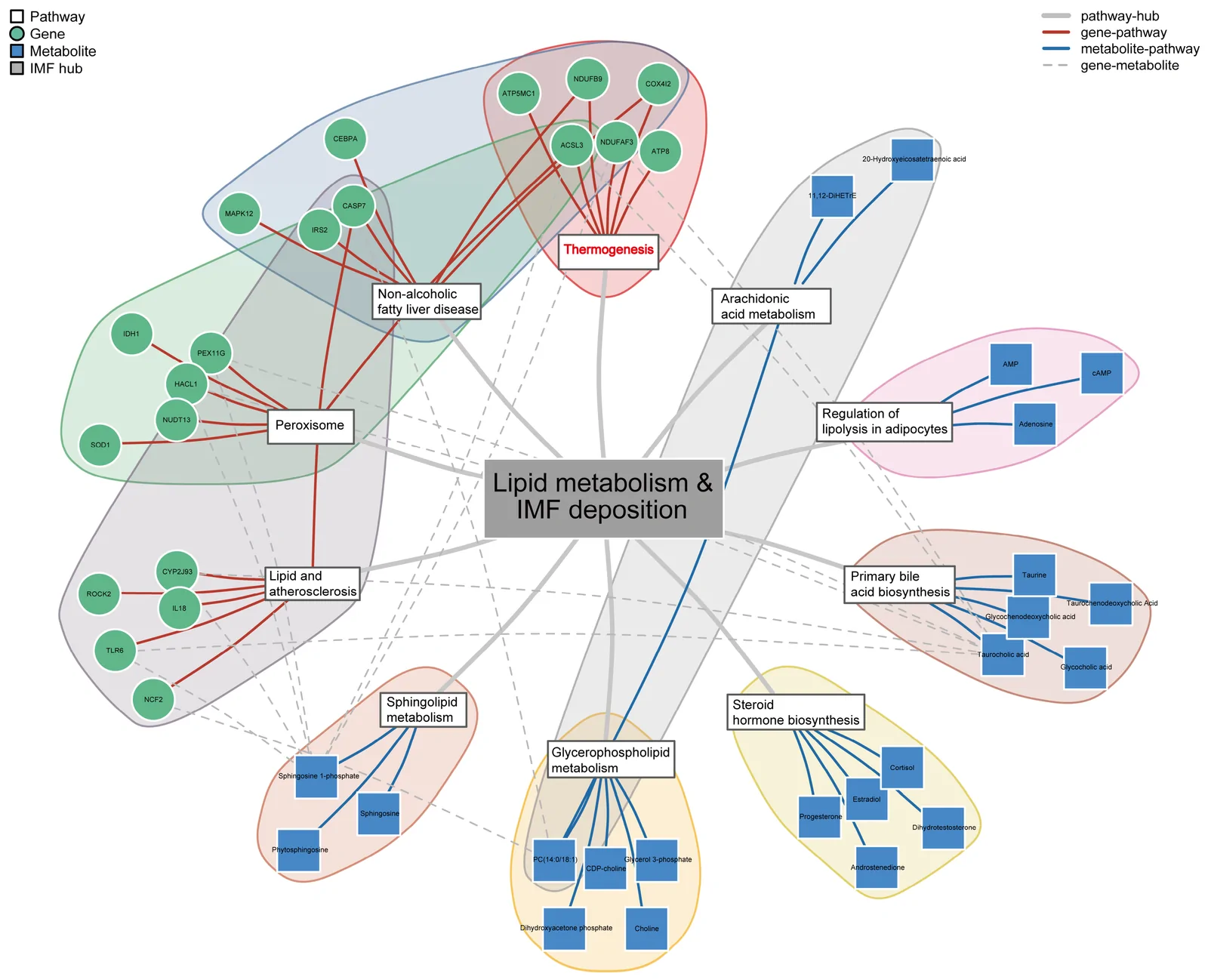
Integrated association (candidate pathway–gene–metabolite) network centered on lipid metabolism and intramuscular fat deposition. Nodes represent KEGG pathways (white), genes (green) and metabolites (blue); gray nodes are IMF hubs; edges denote shared-KEGG-pathway membership (gene–pathway and metabolite–pathway links), i.e., group-level associations rather than experimentally demonstrated regulation. WHZ: Wuhuang pig; WZZ: foreign-breed pig (commercial crossbred pig, Duroc × Landrace × Yorkshire, DLY).

An integrated association (candidate pathway–gene–metabolite) network of “pathway–gene–metabolite” centered on “lipid metabolism and IMF deposition” was constructed (Figure 5): gene modules such as thermogenesis, non-alcoholic fatty liver disease and peroxisome, together with metabolite modules such as sphingolipid metabolism, glycerophospholipid metabolism, steroid hormone biosynthesis and primary bile acid biosynthesis, encircled the center. SCD, FASN, DGAT2, PLIN1, ADIPOQ, THRSP, ELOVL6 and SREBF1 served as the core gene nodes, and sphingosine-1-phosphate (S1P) and taurocholic acid (TCA) served as the metabolic hub molecules, connecting pathways such as sphingolipid metabolism and bile acid metabolism to the central module. The network comprised 31 nodes and 47 edges; S1P was the top metabolite hub (degree = 15), followed by TCA (degree = 10), while SCD (degree = 5) and DGAT2, ADIPOQ, FASN, PLIN1 and THRSP (degree = 4 each) were the principal gene hubs.

### 3.6. Gut Microbiota Identification and Characterization

**Figure 6:**
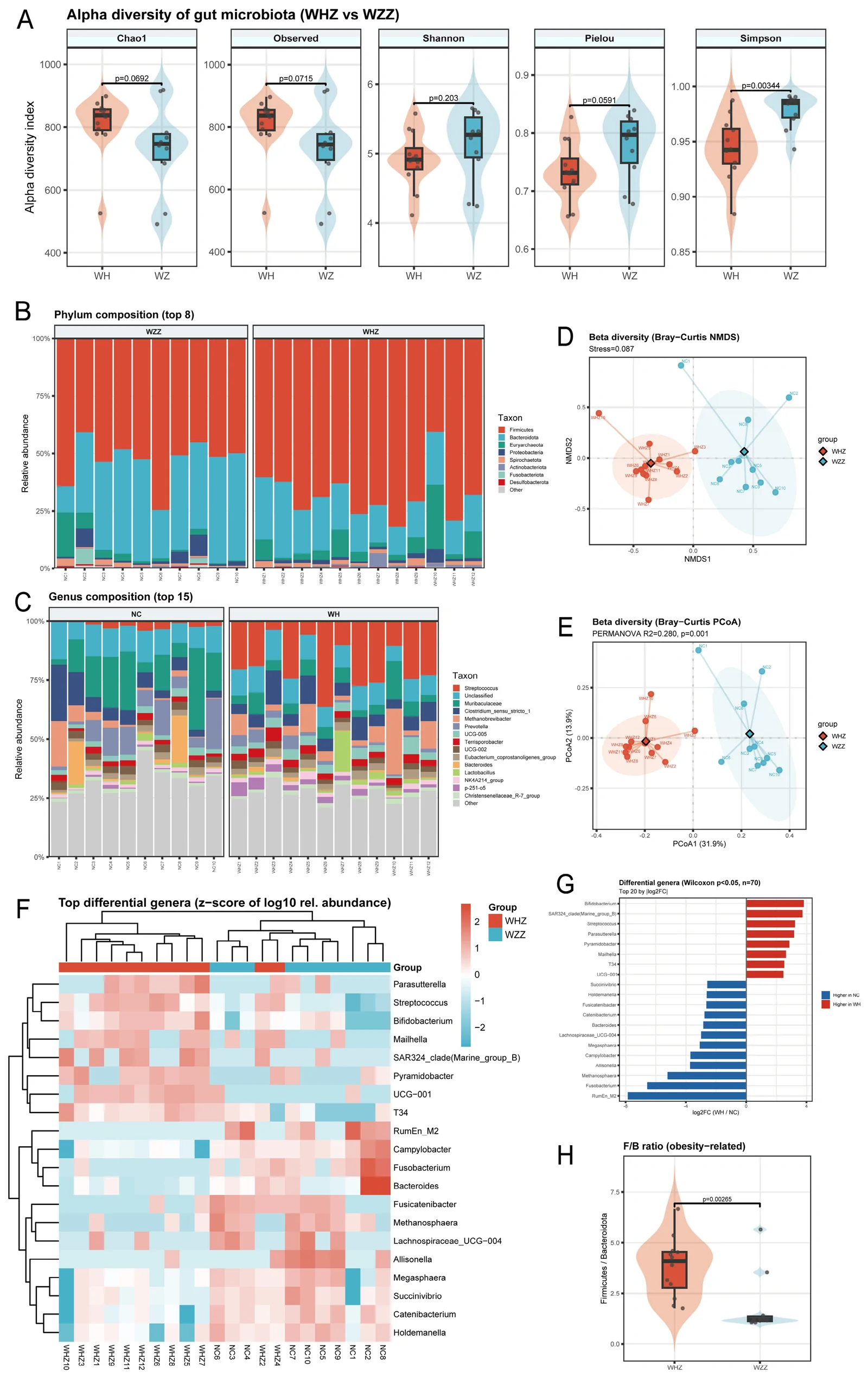
Analysis of gut microbial community structure. (**A**) Comparison of alpha diversity indices (Chao1, observed ASVs, Shannon); (**B**) phylum-level species composition (Top 8; WZZ vs. WHZ); (**C**) genus-level species composition (Top 15); (**D**) Bray–Curtis NMDS analysis (Stress = 0.087); (**E**) Bray–Curtis PCoA analysis (PERMANOVA, R^2^ = 0.28, *p* = 0.001); (**F**) heatmap of the abundance of differential genera (z-score normalized); (**G**) bar plot of differential genera (Wilcoxon *p* < 0.05; the number of differential genera is indicated by n = 70); and (**H**) Firmicutes/Bacteroidetes (F/B) ratio (*p* = 0.00265). WHZ: Wuhuang pig; WZZ: foreign-breed pig (commercial crossbred pig, Duroc × Landrace × Yorkshire, DLY). Fecal 16S rRNA sequencing was performed on n = 12 WHZ and n = 10 WZZ animals after exclusion of one quality-control outlier (WHZ13).

16S rRNA sequencing of the fecal microbiota was performed, and 3701 ASVs were obtained. Alpha diversity analysis showed that the richness of Wuhuang pigs (Chao1, observed ASVs) was slightly higher but not significantly different, whereas the Simpson index was significantly decreased (*p* = 0.0034), suggesting that the microbial community was dominated by a few dominant taxa (Figure 6A). PCoA and NMDS analyses showed clear separation of the two groups along the principal coordinate axes, and the PERMANOVA test confirmed a significant difference in the community structure between the two groups (R^2^ = 0.28, *p* = 0.001, Figure 6D,E). At the phylum level, both groups were dominated by Firmicutes and Bacteroidetes, with Firmicutes accounting for 62.69% and Bacteroidetes for 26.25% in Wuhuang pigs (Figure 6B); the Firmicutes/Bacteroidetes (F/B) ratio was significantly higher than that of foreign-breed pigs (approximately 3.8 vs. 1.9, *p* = 0.00265, Figure 6H), consistent with their stronger energy intake and fat deposition phenotype; the F/B ratio is, however, a coarse, association-level compositional index rather than a causal driver, and it is interpreted accordingly. At the genus level, genera such as *Bifidobacterium*, *Streptococcus* and *Parasutterella* were enriched in Wuhuang pigs (Figure 6C), most of which are associated with short-chain fatty acid production, bile acid metabolism and energy absorption. Using a Wilcoxon *p* < 0.05 threshold, a total of 70 differential genera were identified (Figure 6G), among which *Bifidobacterium* (log2FC = 3.83), *Streptococcus* (log2FC = 3.24) and *Parasutterella* (log2FC = 3.19) were significantly enriched in Wuhuang pigs, whereas the bile-acid-transforming genus *Bacteroides* was depleted (log2FC = −2.84) (Figure 6F), suggesting that gut microbiota may participate in the fat deposition of Wuhuang pigs by promoting energy intake and lipid metabolism.

## 4. Discussion

This study systematically compared the carcass performance and meat quality of Wuhuang pigs and foreign-breed pigs. The crude fat content of Wuhuang pigs reached 6.40%, which was 288% higher than that of foreign-breed pigs, consistent with the performance of fat-type local breeds such as Ningxiang, Rongchang and Jinhua pigs [1,24], and their IMF content (6.40%) was higher than the reported levels of most local pig breeds (2–4%) [1,25]. The muscle fiber diameter, area and density were all lower than those of foreign-breed pigs, similar to the results of studies on local pig breeds such as Mashen and Laiwu pigs [26], and finer muscle fibers are generally associated with better tenderness [27]. In parallel, WHZ pork exhibited a significantly lower pressing loss and a numerically lower shear force, together with favorable postmortem pH and color traits (Table 3); together with the high IMF, these measurable attributes underpin the tenderness, juiciness and flavor that consumers perceive in Wuhuang pork as a food product [25].

Transcriptomic analysis revealed that the upregulation of genes related to lipogenesis and adipocyte differentiation is a prominent transcriptional feature of Wuhuang pigs. The key enzymes of the de novo fatty acid synthesis and triglyceride deposition pathways, such as SCD, FASN, DGAT2 and ELOVL6 [28,29] were coordinately upregulated; SREBF1 (SREBP-1c), as a core transcription factor, can activate the expression of lipogenic genes [30]; THRSP has been reported as a marker of porcine intramuscular adipocytes [31], and the upregulation of PLIN1 and ADIPOQ suggests active lipid droplet storage and adipokine secretion [28]. In addition, the peroxisome and thermogenesis pathways were also enriched, suggesting differences between the two groups in lipid oxidation and energy metabolism. The central role of the SCD–FASN–DGAT2 axis observed here is consistent with integrated transcriptomic/lipidomic evidence from other fatty-type breeds, including Laiwu and Jinhua pigs [11,32], supporting its conserved function in porcine IMF deposition.

Metabolomic analysis confirmed the active state of lipid metabolism in Wuhuang pigs. As a key signaling molecule of sphingolipid metabolism, S1P can activate downstream pathways through S1PR1-5 to participate in adipocyte differentiation and lipid synthesis [30,33]; the de novo sphingolipid synthesis pathway plays an important role in lipogenesis [34]. The elevated acylcarnitine levels suggest relatively active fatty acid oxidation. Bile acids such as taurocholic acid can regulate lipid metabolism through the FXR/TGR5 receptors [35], and the changes in bile acid levels observed in this study correspond to the enrichment of related genera in the microbiota. Taurocholic acid and short-chain fatty acids are themselves gut-microbial products: bacterial bile-salt hydrolases and microbial fermentation shape their circulating pools, providing a plausible route through which the enriched fecal taxa could influence intramuscular lipid composition and, ultimately, the flavor-precursor profile of Wuhuang pork.

Gut microbiota analysis showed that the F/B ratio of Wuhuang pigs was significantly elevated (approximately 3.8 vs. 1.9), consistent with observations in obese populations and animal models [36,37]; the decreased Simpson index indicated a community structure dominated by dominant taxa [13,36]. Fecal microbiota transplantation studies have shown that gut microbiota can promote muscle fat deposition in recipients [2,12,38]. Integrating these correlative multi-omics results, we instead propose a model in which an elevated F/B ratio and an SCFA/bile-acid-oriented microbiota may be associated with greater energy and signaling supply, while host lipogenic genes activate sphingolipid, glycerophospholipid, steroid and bile-acid metabolism to favor IMF deposition [12,39]. Because breed genetics, diet, age, sex and housing can jointly shape IMF and the gut microbiota, and these factors were controlled but not independently manipulated in the present study, their relative contributions cannot yet be separated and require targeted experiments. The core regulatory genes (SCD, FASN, DGAT2, SREBF1, etc.) have been identified as core regulators of fat deposition in pigs in multiple studies [9,11,28,32], indicating the conservation of their regulatory roles. Collectively, these consistent results across the phenotype, transcriptome, metabolome and gut microbiota provide a set of candidate genes, metabolites and taxa that may support local-breed conservation and molecular breeding.

In summary, this multi-omics study characterizes high IMF deposition in Wuhuang pigs across the phenotype, transcriptome, metabolome and gut microbiota. The breed shows clear germplasm advantages in meat quality, with a crude fat content of 6.40% accompanied by finer muscle fibers and better water-holding capacity; at the molecular level, core lipogenic genes (SCD, FASN, DGAT2 and SREBF1) were upregulated and lipid metabolites such as sphingolipids, fatty acids, acylcarnitines and bile acids accumulated, with sphingosine-1-phosphate and taurocholic acid acting as hubs of the lipid-metabolism network. Together, these results provide a set of concrete candidate genes, metabolites and microbial taxa that can support the conservation and utilization of Wuhuang germplasm resources and the genetic improvement of meat quality in local pig breeds. Nevertheless, this study still has certain limitations. First, the sample size, and particularly the number of biological replicates in the RNA-seq analysis (n = 3 per group), was small; the transcriptomic comparison therefore has limited power to detect genes with modest expression changes, and the ranking of marginally significant genes should be regarded as indicative rather than definitive, so that these results are best read as candidates for further validation. Second, because Wuhuang and foreign-breed pigs differ not only in breed but also in body weight at slaughter and growth rate, the observed differences in carcass traits, muscle fiber characteristics and meat quality reflect the combined effects of these factors rather than a pure breed effect, and the results may not be directly transferable to other populations, diets or management systems. The causal relationship between gut microbiota and IMF also remains to be verified by functional experiments such as fecal microbiota transplantation, and only a single muscle site, the longissimus dorsi, was analysed. In the future, spatial transcriptomics, single-cell sequencing and dynamic studies across different growth stages can be combined to further decipher the molecular mechanisms of high IMF formation in Wuhuang pigs.

## 5. Conclusions

Through integrative multi-omics analysis of the phenotype, transcriptome, metabolome and gut microbiome, this study systematically revealed the germplasm advantages of Wuhuang pigs and the molecular basis of their high IMF formation: Wuhuang pigs exhibit typical characteristics of a fat-type pig breed, with a crude fat content of 6.40%; core lipogenic genes such as SCD, FASN, DGAT2 and SREBF1 were upregulated, lipid metabolites including sphingolipids, fatty acids, acylcarnitines and bile acids accumulated, and sphingosine-1-phosphate and taurocholic acid served as the key hubs of the lipid metabolism network; the gut microbiota F/B ratio was elevated, and 70 differential genera were enriched in taxa related to short-chain fatty acid and bile acid metabolism. Together, these correlated layers are consistent with a model in which the “gut microbiota–host metabolism–gene expression–fat deposition” axis may favor high IMF, although causality remains to be established. Overall, this multi-omics analysis provides a theoretical basis and concrete candidate genes, metabolites and microbial taxa for the conservation and utilization of Wuhuang germplasm resources and for genetic improvement of meat quality in local pig breeds. Given the observational design, these candidate, association-level findings should be verified in larger independent cohorts and by functional experiments.

## Figures and Tables

**Table 1:** Primer sequences of the genes used for RT-qPCR validation.

Gene Symbol	Forward Primer	Reverse Primer
MSTN	CCAGAGAGATGACAGCAGTGATG	TTCCTTCCACTTGCATTAGAAGATC
APOE	CTGTGGGTTGCTTTGGTGGT	GCAGGTAATCCCAGAAGCGG
CKM	GTCAGTGTCGCCTCCAGGATA	GTCCCGCAGCTTCTTGTAGA
SOD2	CGCTGAAAAAGGGTGATGTT	AGCGGTCAACTTCTCCTTGA
TNNC1	ATGGTTCGGTGCATGAAGGA	ATCCTCTGTGATGGTCTCGC
TNNI1	CCCCACAGTCTGCAGTCCA	CTCTCAACTTCCGGCATGGT

**Table 2:** Comparison of carcass characteristics between Wuhuang pigs and foreign-breed pigs.

Item	WZZ (n = 14)	WHZ (n = 6)	*p*-Value
Body weight before slaughter (kg)	112.79 ± 4.68	75.17 ± 9.30	<0.001
Carcass weight (kg)	84.43 ± 4.04	52.47 ± 5.81	<0.001
Dressing percentage (%)	74.85 ± 1.38	63.82 ± 0.03	<0.001
Average backfat thickness (cm)	2.10 ± 0.41	3.05 ± 0.41	<0.001
Loin eye area (cm^2^)	52.35 ± 5.82	24.14 ± 4.25	<0.001
Lean meat percentage (%)	63.46 ± 2.66	36.78 ± 0.05	<0.001
Skin and fat percentage (%)	24.87 ± 3.37	44.87 ± 3.82	<0.001
Bone percentage (%)	11.60 ± 1.04	14.53 ± 0.03	0.038

Note: Data are expressed as mean ± SD; *p* < 0.05 indicates a significant difference, and *p* < 0.001 indicates a significant difference. WHZ: Wuhuang pig; WZZ: foreign-breed pig (commercial crossbred pig, Duroc × Landrace × Yorkshire, DLY). SD (not SEM) is shown; each animal contributed one independent measurement (biological replicate), with no technical pseudoreplication.

**Table 3:** Comparison of meat quality between Wuhuang pigs and foreign-breed pigs.

Item	WZZ (n = 7)	WHZ (n = 6)	*p*-Value
pH45min	6.07 ± 0.39	6.03 ± 0.32	0.846
pH24h	5.56 ± 0.08	5.55 ± 0.14	0.871
L45min	42.88 ± 1.11	36.89 ± 2.91	0.003
L24h	55.09 ± 2.75	50.46 ± 3.35	0.019
Pressing loss (%)	30.98 ± 0.02	21.10 ± 0.04	<0.001
Cooking yield (%)	65.32 ± 0.03	66.78 ± 0.02	0.350
Shear force (kg)	6.40 ± 1.94	5.77 ± 1.57	0.536
Crude fat (%)	1.65 ± 0.86	6.40 ± 1.31	<0.001

Note: Data are expressed as mean ± SD; *p* < 0.05 indicates a significant difference, and *p* < 0.001 indicates a significant difference. WHZ: Wuhuang pig; WZZ: foreign-breed pig (commercial crossbred pig, Duroc × Landrace × Yorkshire, DLY). pH45min/pH24h: pH at 45 min/24 h postmortem; L45min/L24h: CIE L* (lightness) at 45 min/24 h postmortem. SD (not SEM) is shown; each animal contributed one independent measurement.

## Data Availability

The original contributions presented in this study are included in the article and Supplementary Material. Further inquiries can be directed to the corresponding authors.

## Supplementary Materials

The following supporting information can be downloaded at https://www.mdpi.com/article/10.3390/foods15183338/s1: Figure S1: OPLS-DA model validation for the muscle metabolome (WZZ vs WHZ). (A) Unsupervised PCA score plot; the WZZ and WHZ samples separate clearly along the principal axes with tight within-group clustering of biological replicates. (B) Permutation test with 1000 random permutations of the class labels; the R2Y and Q2 regression intercepts are shown. (C) OPLS-DA S-plot, highlighting the key differential metabolites (VIP ≥ 1) that drive the separation and their abundance trends in the two groups; Table S1: Sample-level alpha-diversity indices of the fecal microbiota (16S rRNA sequencing).
