# Supplementary material for: Integrative Multi-Omics Analysis Reveals Germplasm Advantages and Candidate Mechanisms Underlying High Intramuscular Fat Deposition in Wuhuang Pigs
Source: Foods. 2026 Sep 20;15(18):3338. doi: 10.3390/foods15183338 (PMC13606551; doi:10.3390/foods15183338)
Supplement: Supplementary file 1 [file foods-15-03338-s001.zip › Supplementary_Material.pdf]

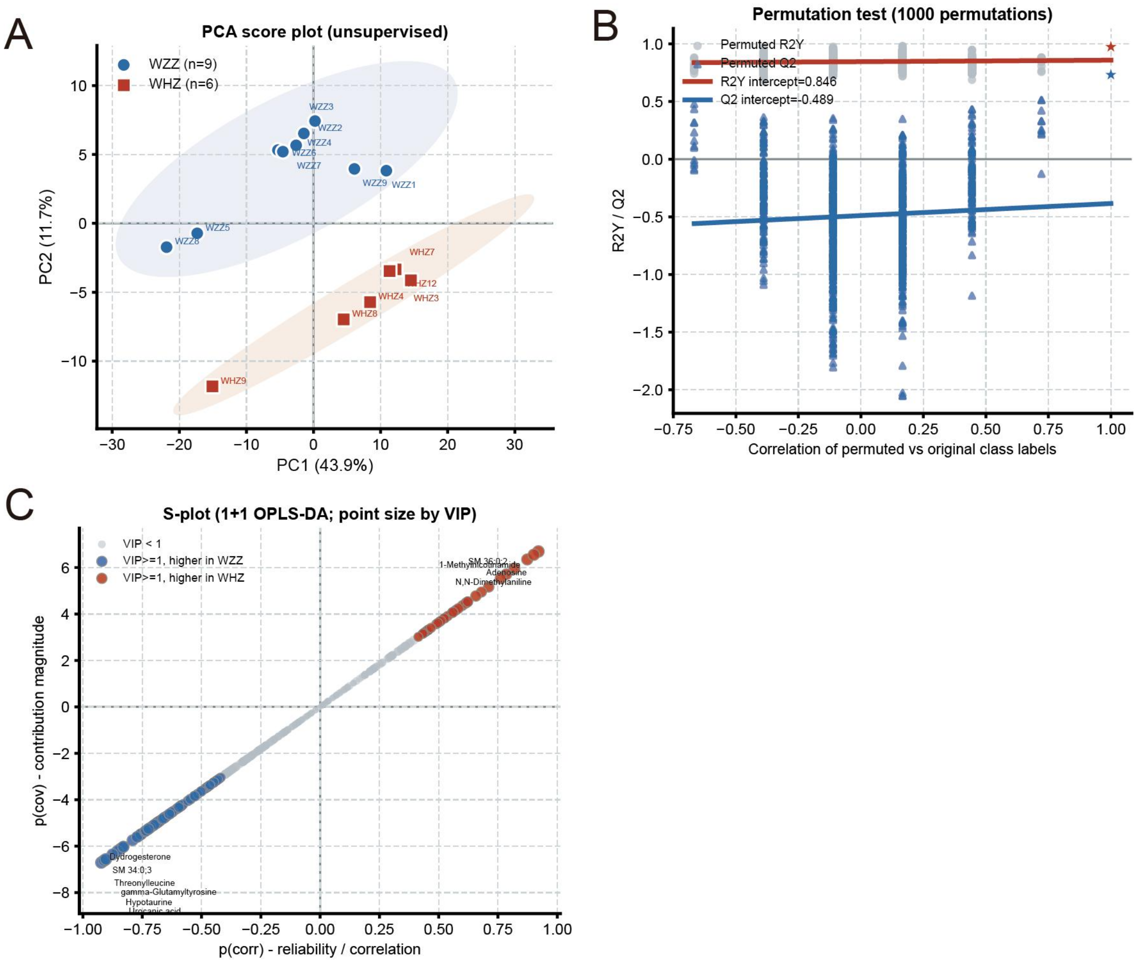

Supplementary Figure S1. OPLS-DA model validation for the muscle metabolome (WZZ vs WHZ). **(A)** Unsupervised PCA score plot; the WZZ and WHZ samples separate clearly along the principal axes with tight within-group clustering of biological replicates. **(B)** Permutation test with 1,000 random permutations of the class labels; the R2Y and Q2 regression intercepts are shown. **(C)** OPLS-DA S-plot, highlighting the key differential metabolites (VIP ≥ 1) that drive the separation and their abundance trends in the two groups.

Supplementary Table S1. Sample-level alpha-diversity indices of the fecal microbiota (16S rRNA sequencing).

Sample	Group	Chao1	Observed ASVs	Shannon	Simpson	Pielou evenness	Dominance	Goods coverage
WHZ1	WHZ	793.45	793	7.06	0.95	0.73	0.05	1.0
WHZ2	WHZ	875.39	875	7.72	0.96	0.79	0.04	1.0
WHZ3	WHZ	842.25	842	8.04	0.99	0.83	0.01	1.0
WHZ4	WHZ	812.61	812	7.2	0.94	0.75	0.06	1.0
WHZ5	WHZ	852.61	851	7.69	0.98	0.79	0.02	1.0
WHZ6	WHZ	775.47	774	6.33	0.88	0.66	0.12	1.0
WHZ7	WHZ	854.52	853	7.17	0.96	0.74	0.04	1.0
WHZ8	WHZ	832.88	832	6.92	0.93	0.71	0.07	1.0
WHZ9	WHZ	898.56	897	7.04	0.93	0.72	0.07	1.0
WHZ10	WHZ	524.84	524	5.93	0.92	0.66	0.08	1.0
WHZ11	WHZ	860.92	860	7.12	0.94	0.73	0.06	1.0
WHZ12	WHZ	778.78	778	6.78	0.94	0.71	0.06	1.0
WHZ13	WHZ	596.73	594	4.82	0.77	0.52	0.23	1.0
NC1	WZZ	524.22	524	6.12	0.94	0.68	0.06	1.0
NC2	WZZ	490.62	489	6.16	0.96	0.69	0.04	1.0
NC3	WZZ	782	782	8.06	0.99	0.84	0.01	1
NC4	WZZ	732.08	731	7.68	0.99	0.81	0.01	1.0
NC5	WZZ	740.2	738	7.54	0.98	0.79	0.02	1.0
NC6	WZZ	918.12	918	8.11	0.99	0.82	0.01	1.0
NC7	WZZ	914.2	913	8.15	0.99	0.83	0.01	1.0

NC8	WZZ	684.08	683	7.24	0.97	0.77	0.03	1.0
NC9	WZZ	766.26	764	7.1	0.97	0.74	0.03	1.0
NC10	WZZ	751.61	750	7.67	0.99	0.8	0.01	1.0
WHZ (n = 12, excluding WHZ13)		808.52 ± 93.01	807.58 ± 92.95	7.08 ± 0.56	0.94 ± 0.03	0.73 ± 0.05	0.06 ± 0.03	1.00 ± 0.00
WZZ (n = 10)		730.34 ± 132.54	729.20 ± 132.62	7.38 ± 0.70	0.98 ± 0.01	0.78 ± 0.05	0.02 ± 0.01	1.00 ± 0.00
WHZ13 (excluded)		596.73 ± 0.00	594.00 ± 0.00	4.82 ± 0.00	0.77 ± 0.00	0.52 ± 0.00	0.23 ± 0.00	1.00 ± 0.00

Note: WHZ, Wuhuang pigs; WZZ, foreign-breed control (NC in the raw data). Samples were rarefied to an equal sequencing depth before alpha-diversity calculation; the outlier sample WHZ13 was excluded from the group comparison (see main text).
